# Interleukin-6 Receptor Antibodies for Modulating the Systemic Inflammatory Response after Out-of-Hospital Cardiac Arrest (IMICA): study protocol for a double-blinded, placebo-controlled, single-center, randomized clinical trial

**DOI:** 10.1186/s13063-020-04783-4

**Published:** 2020-10-20

**Authors:** Martin A. S. Meyer, Sebastian Wiberg, Johannes Grand, Jesper Kjaergaard, Christian Hassager

**Affiliations:** Department of Cardiology, The Heart Centre, Copenhagen University Hospital – Rigshospitalet, Copenhagen, Denmark

**Keywords:** Randomized controlled trial, Out-of-hospital cardiac arrest, Systemic inflammation, Post-cardiac arrest syndrome, Interleukin-6, Tocilizumab, Organ damage, Hemodynamics

## Abstract

**Background:**

Resuscitated out-of-hospital cardiac arrest (OHCA) patients who remain comatose at admission are at high risk of morbidity and mortality. This has been attributed to the post-cardiac arrest syndrome (PCAS) which encompasses multiple interacting components, including systemic inflammation. Elevated levels of circulating interleukin-6 (IL-6), a pro-inflammatory cytokine, is associated with worse outcomes in OHCA patients, including higher vasopressor requirements and higher mortality rates. In this study, we aim to reduce systemic inflammation after OHCA by administering a single infusion of tocilizumab, an IL-6 receptor antibody approved for use for other indications.

**Methods:**

Investigator-initiated, double-blinded, placebo-controlled, single-center, randomized clinical trial in comatose OHCA patients admitted to an intensive cardiac care unit. Brief inclusion criteria: OHCA of presumed cardiac cause, persistent unconsciousness, age ≥ 18 years. Intervention: 80 patients will be randomized in a 1:1 ratio to a single 1-h intravenous infusion of either tocilizumab or placebo (NaCl). During the study period, patients will receive standard of care, including sedation and targeted temperature management of 36 ° for at least 24 h, vasopressors and/or inotropes as/if needed, prophylactic antibiotics, and any additional treatment at the discretion of the treating physician. Blood samples are drawn for measurements of biomarkers included in the primary and secondary endpoints during the initial 72 h. Primary endpoint: reduction in C-reactive protein (CRP). Secondary endpoints (abbreviated): cytokine levels, markers of brain, cardiac, kidney and liver damage, hemodynamic and hemostatic function, adverse events, and follow-up assessment of cerebral function and mortality.

**Discussion:**

We hypothesize that reducing the effect of circulating IL-6 by administering an IL-6 receptor antibody will mitigate the systemic inflammatory response and thereby modify the severity of PCAS, in turn leading to lessened vasopressor use, more normal hemodynamics, and better organ function. This will be assessed by primarily focusing on hemodynamics and biomarkers of organ damage during the initial 72 h.

In addition, pro-inflammatory and anti-inflammatory cytokines will be measured to assess if cytokine patterns are modulated by IL-6 receptor blockage.

**Trial registration:**

ClinicalTrials.gov Identifier: NCT03863015; submitted February 22, 2019, first posted March 5, 2019.

EudraCT: 2018-002686-19; date study was authorized to proceed: November 7, 2018.

## Administrative information

The order of the items has been modified to group similar items (see http://www.equator-network.org/reporting-guidelines/spirit-2013-statement-defining-standard-protocol-items-for-clinical-trials/).

**Table:** 

Title {1}	Interleukin-6 Receptor Antibodies for Modulating the Systemic Inflammatory Response after Out-of-Hospital Cardiac Arrest (IMICA): study protocol for a double-blinded, placebo-controlled, single-center, randomized clinical trial
Trial registration {2a and 2b}.	ClinicalTrials.gov Identifier: NCT03863015 EudraCT: 2018-002686-19
Protocol version {3}	Version: 1.91 of November 1, 2019 (Minor updates to v. 1.9 of October 25, 2018)
Funding {4}	Hjertecenterets Forskningsudvalg (The Heart Center Research Council, Rigshospitalet): The cost of establishing the biobank. Hjerteforeningen (Danish Heart Foundation): Funding of salary for MASM in 1 year and funding of analysis of biomarkers from the biobank, the cost of personnel involved in the MR substudy, as well as covering costs for presentations of the study results at conferences. Region Hovedstadens Forskningsfond til sundhedsforskning, Denmark (Captial Region Research foundation): Salary for MASM NovoNordisk Foundation: JK is supported by an unrestricted grant from the NovoNordisk Foundation (grant NNF17OC0028706) for research in post-cardiac arrest management.
Author details {5a}	All authors: Dept. of Cardiology, The Heart Centre, Copenhagen University Hospital - Rigshospitalet, Copenhagen, Denmark
Name and contact information for the trial sponsor {5b}	Trial sponsor-investigator: Christian Hassager, Professor, MD, DMSc Dept. of Cardiology, The Heart Centre, Copenhagen University Hospital - Rigshospitalet, Copenhagen, Denmark Email: Christian.hassager@regionh.dk
Role of sponsor {5c}	This is a sponsor-investigator-initiated study with no funding or involvement from pharmaceutical companies, and the sponsor-investigator maintains authority over all aspects of the trial including, design, management, interpretation of results, and publication.

## Introduction

### Background and rationale {6a}

Resuscitated out-of-hospital cardiac arrest (OHCA) patients who remain comatose at admission are at high risk of morbidity, and mortality remains above 50% at 30 days [1]. Consequently, an increasing emphasis on post-resuscitation care has been addressed by current guidelines [2, 3].

The high mortality in comatose OHCA patients has been attributed to the post-cardiac arrest syndrome (PCAS), which includes four mutually interacting components: systemic ischemia/reperfusion response, cerebral injury, myocardial dysfunction, and the persistent precipitating cause of the arrest [4]. Despite repeated emphasis on post-resuscitation care, no specific therapies targeting PCAS have been implemented except targeted temperature management (TTM), which has been recommended since 2003 [5]. Thus, research addressing mitigation of the PCAS is warranted. During OHCA, patients are exposed to whole-body ischemia, which is ensued by reperfusion injury after resuscitation, both of which trigger activation of inflammatory cascades leading to a systemic inflammatory response or sepsis-like syndrome [6–9]. High levels of Inflammatory markers, including procalcitonin (PCT) [9–11], C-reactive protein (CRP) [9], interleukin (IL)-6 [9], and IL-10 [9], have been associated with unfavorable outcome after OHCA. Furthermore, the inflammatory markers interleukin-1β (IL-1β), IL-6, IL-10, and tumor necrosis factor α (TNF-α) have all been associated with the severity of PCAS, assessed by sequential organ failure assessment (SOFA) score in unconscious OHCA patients [8]. Importantly, levels of IL-6 have been shown to be independently associated with poor outcomes in unconscious OHCA patients after adjustment for known risk markers [9]. Further, the level of IL-6 was more strongly associated with PCAS severity compared to classical inflammatory markers such as CRP and PCT [8].

IL-6 is a pro-inflammatory cytokine secreted by T cells and macrophages. IL-6 readily crosses the blood-brain barrier [12] and is a mediator of fever. Further, IL-6 is a mediator of the acute phase response and plays a role in activation of the coagulation system, increasing vascular permeability and weakening papillary muscle contractions leading to myocardial dysfunction [13]. IL-6 has been found to be involved in a range of pathological processes including tissue hypoxia, disseminated intravascular coagulation (DIC), and multiorgan failure [13], all of which represent parts of the SIRS response. IL-6 has been suggested to play a role in ischemia-reperfusion injury in myocardial infarction (MI), and higher levels of IL-6 have been associated both with the magnitude of myocardial injury, mortality, and adverse events in this group [14–16].

Due to the role of IL-6 in many inflammatory diseases, IL-6 receptor antibodies (IL-6RA) have been developed. The first IL-6RA, tocilizumab, was approved for treatment of rheumatoid arthritis in 2009 and has later been approved for giant cell arthritis and chimeric antigen receptor (CAR) T cell-induced cytokine release syndrome. In addition to the approved indications, tocilizumab has been suggested to have other beneficial immune-modulating and organ-protective effects against the autoimmune neurological disorders neuromyelitis optica and autoimmune encephalitis [17–20]. In patients presenting with non-ST-elevation myocardial infarction (NSTEMI), a 1-h infusion of 280 mg tocilizumab decreased the inflammatory response assessed by CRP levels and further decreased myocardial injury assessed by TnT levels [21]; importantly, no increased risk of adverse events was observed in patients receiving tocilizumab. Animal data suggest that tocilizumab is safe and effective for treatment of severe acute pancreatitis and associated acute lung injury [22]. Further, tocilizumab had neuroprotective effects in a model of Alzheimer’s disease [23].

In summary, resuscitated OHCA is associated with a systemic inflammatory response, the magnitude of which has been associated with increased mortality and poor neurological outcome. IL6 was associated with the severity of PCAS and mortality in the large Target Temperature Management (TTM) Trial in OHCA patients. Further, the decision to target IL-6 receptor blockage in the present trial was also based on the finding that while IL-6 measured as early as at admission—where the intervention would commence—was associated with the degree of PCAS on the days following admission, this was not the case for other potential pharmaceutical targets in the pro-inflammatory pathways such as IL-1β or TNF-α [8]. Inhibiting the IL-6-mediated immune response by infusion of the IL6RA tocilizumab in the acute phase after hospital admission may mitigate the systemic inflammatory response and may further inhibit ischemia-reperfusion injury possibly leading to improved outcomes.

### Objectives {7}

#### Primary objective

The primary objective of this study is to determine the efficacy of an interleukin-6 (IL-6) receptor antibody (IL-6RA) compared with placebo on the endpoint of daily high-sensitivity c-reactive protein (hsCRP) measurements from admission (i.e., prior to initiation of the study drug) to the first 72 h after admission in comatose patients admitted after resuscitated OHCA.

#### Secondary objective

The secondary objectives of this study are to determine the effects of an IL-6RA on inhibition of inflammation, cardiac protection, neuroprotection, renal protection, endothelial protection, clinical endpoints including survival, and neurological outcome, as well as safety.

#### Hypothesis

A 1-h infusion of the IL-6RA tocilizumab initiated as soon as possible after return of spontaneous circulation (ROSC) will reduce the SIRS-like response assessed by hsCRP levels in unconscious OHCA patients.

### Trial design {8}

This is an investigator-initiated, randomized, placebo-controlled, double-blinded clinical phase II trial. Following successful completion of screening procedures, subjects will be randomized in a 1:1 fashion to receive IL-6RA or placebo.

## Methods: participants, interventions, and outcomes

### Study setting {9}

It is a single-center study carried out at the Cardiac Intensive Care Unit within the Department of Cardiology, The Heart Centre, Copenhagen University Hospital - Rigshospitalet, Copenhagen.

### Eligibility criteria {10}

From trial initiation, a screening log will be completed on a day-to-day basis including limited information about each potential study candidate in accordance with current legislation, date of admission, and outcome of the screening (i.e., enrolled, reasons for ineligibility, and refusal to participate). Screening will be performed by the physician on call or a dedicated team of research personnel, all medical doctors. After inclusion in the study by a qualified medical doctor, infusion of the study drug will be managed by the attending ICU nurse.

Inclusion in the IMICA trial does not prohibit participation in other trials, and the vast majority of patients are also expected to be enrolled in the multi-center randomized trial “Blood Pressure and OXygenation Targets After OHCA (BOX),” which investigates the optimum targets for blood pressure and oxygenation after OHCA, and investigate an automated feedback temperature control-device for fever-control duration of targeted temperature management; ClinicalTrial.gov identifier: NCT03141099.

### Inclusion criteria

Each of the following criteria must be fulfilled for a subject to be eligible:

1. Age ≥ 18 years
2. OHCA of a presumed cardiac cause as assessed by the treating physician at the time of admission
3. Unconsciousness upon admission, i.e., a GCS < 9
4. Sustained ROSC for more than 20 min

### Exclusion criteria

None of the following criteria must be fulfilled for a subject to be eligible:

1. Unwitnessed asystole
2. Suspected or confirmed intracranial bleeding or stroke
3. Pregnancy, or females in fertile age, unless a negative serum HCG can rule out pregnancy within the inclusion window.
4. Temperature on admission < 30 °C
5. Persistent cardiogenic shock (defined as systolic blood pressure (SBP) < 90 despite relevant fluid resuscitation, vasopressor, and inotropic support) that is not reversed within the inclusion window (patient can be randomized if SBP recovers within this window).
6. Any known disease making 180-day survival unlikely
7. Known limitations in therapy, i.e., a do-not-resuscitate order or a decision to withhold critical care
8. Known pre-arrest Cerebral Performance Category of 3 to 4
9. > 240 min from ROSC to randomization
10. Known allergies to IL-6RA
11. Known infection
12. Known hepatic cirrhosis

### Who will take informed consent? {26a}

The trial will be conducted in accordance with the Declaration of Helsinki and follows EU and national legislation on medical research in subjects in emergency situations being temporarily incapacitated. All subjects are incapacitated according to the inclusion criteria. Thus, subjects will not be able to provide informed consent. National legislation requires proxy consent from a legal surrogate, in this trial, a medical doctor with no involvement in the patient treatment, as well as consent from the next of kin. The legal surrogate will be consented prior to inclusion in the study and the next of kin at the earliest possible time. Consent will be obtained by the physician on call or a dedicated team of research personnel, all medical doctors. Please see the “Ethics approval and consent to participate” {24} section for approvals from the relevant authorities.

### Additional consent provisions for collection and use of participant data and biological specimens {26b}

The use of patient data or biological specimens for ancillary studies will be dependent on approval from the local ethical committee unless this is waived based on prior approvals or the design of the studies.

## Interventions

### Explanation for the choice of comparators {6b}

Aiming for an attenuation of the systemic inflammatory response after OHCA by use of an IL-6RA, tocilizumab, we have chosen to give a single 1-h infusion of tocilizumab 8 mg/kg (maximum 800 mg) as this is the dosage used for repeated infusions in rheumatoid arthritis, an already approved indication [24].

### Intervention description {11a}

Patients being allocated to IL-6RA will receive a 1-h infusion of 8 mg tocilizumab per kilogram body weight (maximum dose 800 mg, i.e., 40 mL of concentrate), equivalent to 0.4 mL per kilogram bodyweight (study drug concentration 20 mg/mL); the study drug will be suspended in normal saline to a total volume of 100 mL. Patients being allocated to placebo will receive a 1-h infusion of 100 mL of normal saline. Hence, for both the active and placebo arm, patients will receive a 1-h infusion of 100 mL. The infusion of either IL-6RA or placebo will commence at the earliest possible time after admission and study inclusion.

### Criteria for discontinuing or modifying allocated interventions {11b}

A subject or the subject’s next of kin has the right to withdraw from the study at any time, for any reason, without prejudice to his or her future medical care by the physician or at the institution. Any subject who withdraws consent to participate in the study will immediately be removed from further treatment and/or study observation on the date of request. In addition, the investigator and the sponsor have the right to withdraw a subject from the study if any of the following occurs:

1. Significant intercurrent illness
2. Refusal by the subject to continue observations
3. Decision by the investigator that termination is in the subject’s best medical interest

Should a subject (or legally acceptable representative) requests or decides to withdraw from the study, all efforts will be made to complete and report the observations as thoroughly as possible up to the date of withdrawal. All information should be reported on the applicable case report forms. A complete final evaluation and assessments should be made at the time of the subject’s withdrawal. The end of study case report form will be completed with an explanation for the withdrawal. If the withdrawal of a subject is due to an adverse event, follow-up visits should be scheduled until the adverse event has been resolved or stabilized. Unless consent has been withdrawn, follow-up data on deaths and hospitalizations will be collected until study termination or a maximum of 48 months since enrollment of the subject. Unless specifically requested by the patient, the patient will be followed for the primary endpoint until the end of the study.

### Strategies to improve adherence to interventions {11c}

The clinical personnel, i.e., nurses and doctors, who are directly involved in the care of the study patients have been trained in the study-specific procedures prior to involvement in the study, and likewise, nurses who prepare the study drug have been trained as appropriate. The study intervention is a single 1-h infusion limiting the risk of missing dosages; however, as the study is carried out in patients who can be inherently unstable, there is a risk of missing or delaying the intervention due to other clinical emergency interventions. To limit this risk, a checklist has been developed for the ICU monitoring procedures and interventions not only related to the study, but also encompasses “standard of care”; also, the study drug is prepared at a cardiac care unit with separate facilities for preparing the study drug, within the Department of Cardiology, who are not engaged in the initial treatment of the study patients.

### Relevant concomitant care permitted or prohibited during the trial {11d}

There are no restrictions in concomitant care for trial participants, patients will be treated with standard therapies for OHCA according to contemporary guidelines, and necessary cardiac interventions will not be delayed by the trial intervention; however, efforts will be made to maintain study drug infusion during treatment.

Specifically, during the study period, patients will receive standard of care, including sedation and targeted temperature management of 36 ° for at least 24 h, vasopressors and/or inotropes as/if needed, prophylactic antibiotics (intravenous piperacillin/tazobactam or cefuroxime in case of beta-lactam allergy), and any additional treatment at the discretion of the treating physician.

### Provisions for post-trial care {30}

Participating subjects will be insured by the health system responsible for the trial site, “Rigshospitalet.”

### Outcomes {12}

#### Primary endpoint

The primary endpoints are measurements of hsCRP at admission and at 24, 48 and 72 h.

#### Secondary endpoints

##### Markers of inflammation

Markers of inflammation are the following:

Daily interleukin cytokine levels (IL-1b, IL-2, IL-4, IL-5, IL-6, IL-7, IL-8 IL-10, IL-12, IL-13, IL-17A, G-CSF, GM-CSF, MCP-1, MIP-1beta, INF-g, and TNF-α IL-1b, IL-6, IL-10, IL-13, TNF-α) from admission to 72 h after admission (analysis of samples in biobank)

Daily leucocyte differential counts the first 3 days from admission

Daily Sequential Organ Failure Assessment (SOFA) scores the first 3 days from admission

##### Markers of cerebral injury

Markers of cerebral injury are neuron-specific enolase (NSE) levels after 48 and 72 h (routine biochemistry) and other markers of cerebral injury (analysis of samples in biobank).

##### Markers of cardiac injury

Markers of cardiac injury are daily high-sensitivity troponin T (TnT hs) and CKMB levels the first 3 days from admission.

##### Markers of hemodynamic function

Markers of hemodynamic function are the following:

Daily Swan-Ganz-based measurements of cardiac output (CO), central venous pressure (CVP), pulmonary capillary wedge pressure (PCWP), and systemic vascular resistance (SVR)

Mean arterial pressure

Bihourly analyses of arterial blood gas the first 36 h

Echocardiography on the day following admission and on either day 3, 4, or 5 Focused examination: systolic function of left and right ventricle, i.e., left ventricular ejection fraction (LVEF) and tricuspid annular plane systolic excursion (TAPSE)

##### Markers of kidney injury

Markers of kidney injury are daily creatinine levels the first 3 days from admission.

##### Markers of hepatic injury

Markers of hepatic injury are daily measurements of ALAT, ASAT, bilirubin, and INR the first 3 days from admission.

##### Markers of endothelial injury

Markers of endothelial injury are daily soluble thrombomodulin levels the first 3 days from admission.

##### Markers of the coagulation system

Markers of the coagulation system are plasma fibrinogen the first 3 days from admission and thrombelastography at admission and 48 h.

#### Clinical endpoints

The clinical endpoints are the following:

Length of ICU and hospital stay

Survival at 30 days, 90 days, 180 days, and at end of trial

Montreal Cognitive Assessment (MOCA) score at 90 days

Cerebral Performance Category (CPC) and modified Rankin scale (mRS) at 30 days, 90 days and 180 days

#### Safety

Cumulated incidence of adverse events is in the first 7 days.

### Participant timeline {13}

See spirit Table 1 and the section containing primary and secondary outcomes above.

**Table 1:** Spirit figure

	Study period							
	Enrollment	Post-allocation						Follow-up
**Timepoint**	**0 h**	**6 h**	**12 h**	**24 h**	**36 h**	**48 h**	**72 h**	**Days 30, 90, and 180**
**Enrollment**								
**Eligibility screen**	x							
**Informed consent**	Legal guardian and next of kin							Patient as soon as possible
**Randomization**	x							
**Interventions**								
**Preparation of study drug and beginning of 1-h infusion**	x							
**Assessments**								
**Biochemestry**^**1**^	x	x	x	x	x	x	x	
**Biobank samples**	x			x		x	x	
**ECG**	x	x	x	x	x	x	x	
**Swan-Ganz based measurements**	x	x	x	x	x	x	x	
**Echocardiography**				Day 1		Either day 3, 4, or 5		
**ABG and VBG**^**2**^	x	x	x	x	x	x	x	
**SOFA score**				x		x	x	
**CPC and mRS score**								x
**MOCA score**								Only day 90

*ECG* electrocardiogram, *ABG* arterial blood gas, *VBG* venous blood gas, *SOFA score* Sequental Organ Failure Assesment, *CPC* Cerebral Performance Category, *MOCA* Montreal Cognitive Assesment

^1^See outcomes section in manuscript for further detail

^2^Additional blood gasses are taken bihourly until 12 h and at 18 h

### Sample size {14}

The trial is powered at the primary endpoint. A previous trial has shown an effect of tocilizumab on hsCRP in NSTEMI patients [21]. That trial demonstrated a median area under the hsCRP curve of 2.0 mg/L/h in patients receiving tocilizumab compared to 4.2 mg/L/h in patients receiving placebo, i.e., a reduction of 52%. However, the systemic inflammatory response in a NSTEMI population will be limited compared to an OHCA population. For example, hsCRP levels in NSTEMI patients have been reported at approximately 2–6 mg/L [21], whereas CRP levels in OHCA patients have been reported at approximately 100–250 mg/L after 48–72 h [25].

As no previous data exists regarding the effect of tocilizumab on hsCRP over time, we chose to power the present trial towards a single hsCRP measurement drawn 48 h after admission. In 140 resuscitated OHCA patients from our institution, the mean hsCRP level after 48 h was 179 ± 75 mg/L (unpublished data). We assumed that tocilicumab treatment would reduce the hsCRP level by 30%. Assuming an α-level of 0.05, the trial would achieve a power of 0.81, if 64 patients were included. However, taking mortality within the first 3 days (estimated at 8% [26]) as well as blood samples missing for other reasons into account, we aimed to include 80 patients.

### Recruitment {15}

The time necessary for including the intended 80 patients is estimated to be 1 year from enrollment of the first patient. This estimate is based on inclusion rates of three previous studies in OHCA patients carried out at the Department of Cardiology, Rigshospitalet [8, 26, 27].

## Assignment of interventions: allocation

### Sequence generation {16a}

Successfully screened subjects will be randomized into the study by the investigator, or assigned co-investigator, using a secure web-based electronic Case Report System (Zenodotus eCRF), developed and hosted by our institutional research department for in-house randomized trials. To allow randomization, the system requires the investigator to enter a valid patient identification number and verify all inclusion and exclusion criteria. A prefabricated allocation sequence is produced using a STATA, Version 13 (StataCorp, TX, USA) script, with random allocation based on the uniform() function, and a fixed seed set to assure reproducibility. This sequence list is then uploaded to the Case Report System, which will use the list to sequentially allocate treatment, as patients are enrolled. The script is set up to allocate treatment in a 1:1 ratio of active treatment or placebo in permuted blocks of four. The generation of the allocation sequence and configuration of the electronic case report form is performed by a senior research physician from our institution, who is otherwise not involved in the trial conduction. All treating physicians and study coordinators will remain blinded to the allocation sequence throughout the trial. The case report form system does however allow emergency unblinding to be performed by the lead study investigators, which in this case automatically will be logged by the system.

### Concealment mechanism {16b}

The treatment assignment is managed by the above-described website and information on a specific treatment assignment is during the trial only available for the nurse who prepares the study drug as described below. After completion of the study drug for each patient, a document with assignment, information on lot-numbers, dosage, and who prepared it is completed and stored in a sealed envelope.

### Implementation {16c}

After randomization of each subject, the doctor who randomized or the attending ICU nurse will contact a prespecified neighboring department and request preparation of the study drug. Only the nurses at the neighboring department who are responsible for preparing the study drug will be able to access the randomization website with specific log-in credentials and see the assigned treatment of either active (IL-6RA / RoActemra) or placebo (NaCl).

The investigator will only after completion of the trial receive a computer-generated code regarding treatment assignment “A” or “B” and at the time of unblinding be informed of which is active/placebo.

## Assignment of interventions: blinding

### Who will be blinded {17a}

Clinical personnel, research personnel, and patients, except for the nurses who prepare the study drug (not involved in patient care, see below), will be blinded to the assigned interventions during the study period. For data analysis, assignments will be labeled “A” and “B,” and only after completion of the calculations will assignments be unblinded.

### Procedure for unblinding if needed {17b}

The identification of treatment allocation will be maintained at a computer located at Rigshospitalet, protected from participants of the study. Authorized site staff will be provided with a unique Personal Identification Number (PIN) to obtain unblinding information.

For each subject, after preparation of the study drug, a document is filled out with information on treatment allocation, lot numbers, and who prepared it as described above; this document is then stored in a concealed envelope and should only be opened after the trial has been completed and unblinding is intended or on a patient-specific basis as described below; these envelopes will also be available for monitoring agencies who will then reseal after monitoring. A subject’s treatment assignment should only be unblinded by a study site when knowledge of the treatment is essential for the further management of the subject or if needed for safety reporting to regulatory authorities. Unblinding at the study site for any other reason will be considered a protocol deviation.

For unblinding at the site, the Principal Investigator should if possible be contacted before unblinding any subject’s treatment assignment. At the latest, the sponsor-investigator must be notified within 1 working day after the event, and the unblinding must be documented in the subject’s case report form.

## Data collection and management

### Plans for assessment and collection of outcomes {18a}

Patient data will be handled as ordinary chart records. All data will be kept according to national legislation. The study database will be constructed in REDCap® in accordance with national legislation and local practice (see “Ethics approval and consent to participate” {24} section for approvals). The database will be maintained for 15 years and anonymized if requested by relevant authorities. Data from the analysis of biomarkers from the biobank will be stored on an approved server with back-up function.

Data entries in RedCap® will be monitored by a Good Clinical Practice (GCP) unit as described below.

### Plans to promote participant retention and complete follow-up {18b}

During the initial 3 days after admission, where the majority of data collection, and all blood sampling, are scheduled, the majority of patients are expected to remain within the Cardiac ICU where the study is harbored; those who experience an expedited recovery and are moved out of the ICU to a neighboring ward will be sought sampled for blood as well; only a minority of patients are expected to be transferred to another hospital within the initial 3 days; however, these patients cannot be sampled for the biobank, yet any routine biochemistry, including CRP, can be accessed by use of a shared electronic medical file. For follow-up, the contact information for patients and their next of kin is stored with the intention of inviting patients to a “90 days follow-up evaluation” at Rigshospitalet. Those who cannot or will not come to the hospital will receive a follow-up questionnaire by letter. Finally, after 180 days, the patients or next of kin will be contacted by phone for a brief cognitive evaluation; both the regional medical file and the joint national medical file will be used for evaluation as well.

### Data management {19}

See the “Plans for assessment and collection of outcomes {18a}” section above.

### Confidentiality {27}

As listed above, all data will be kept in accordance with the approved data handling plan (approved by the Legal Department of Rigshospitalet, approval id VD-2019-26), ensuring adherence to relevant national and international legislation. Data from ordinary medical charts will be entered in REDCap®, and results from analysis of biobank material will be kept on a secured server. Data will only be shared based on approval from the relevant authorities.

### Plans for collection, laboratory evaluation and storage of biological specimens for genetic or molecular analysis in this trial/future use {33}

A biobank will be kept according to the granted approvals, and planned analyses as listed in the “Outcomes {12}” section will be carried out at an experienced laboratory. Future studies will be able to access this pertinent on approval from relevant authorities. At this time, no genetic studies are planned.

## Statistical methods

### Statistical methods for primary and secondary outcomes {20a}

All analyses will be conducted on the modified intention-to-treat population with a set significance level of *p* < 0.05. When interpreting results, confidence intervals, and not only *p* values, will be considered. Throughout, categorical variables will be presented as numbers (frequencies), whereas continuous variables will be presented as mean ± standard deviation (SD) if normally distributed and as median (25th percentile–75th percentile) if non-normally distributed.

#### Analysis of continuous endpoints

Continuous endpoints being assessed at multiple timepoints (including the primary endpoint) will, based on measurements for each timepoint, be analyzed by application of linear mixed models of covariance. The main results of these analyses will be the treatment-by-time interaction as a marker of whether the individual endpoint changes differently over time in the tocilizumab versus the placebo arm and for endpoints where sampling is done only after the intervention has been ongoing for a prolonged time and no baseline sample exists (e.g., NSE which is measured at 48 h and 72 h); also, group effects will be considered. As admission blood samples are scheduled to be drawn prior to study infusion, baseline correction will be employed as appropriate. Logarithmic transformation will be applied to approximate normal distribution as appropriate. The application of linear mixed models of covariance for analysis of the primary endpoint will result in a somewhat higher power as compared to the sample size calculation (see above) being based on a single measurement. As many trials apply a power of 0.90 for sample size calculations, we deem this acceptable. In case of missingness greater than 5% for the primary endpoint, multiple imputations by chained equations will be applied as sensitivity analysis with generation of 20 individual data sets.

#### Analysis of categorical endpoints

Categorical endpoints by treatment allocation will be analyzed by the chi-squared or Fisher’s exact test, as appropriate.

#### Analysis of survival data

Kaplan-Meier curves for each allocation group will be estimated, graphically displayed, and compared by the log-rank test. Further Cox proportional hazard models will be applied to assess differences in time to death between treatment groups. These models will sequentially be adjusted for the interaction between treatment allocation and each of the following variables: sex, age, time to ROSC, lactate level upon admission, shockable primary rhythm, STEMI upon admission, pPCI performed, and levels of inflammatory markers including IL-6 and hsCRP. Further, models stratified by cause of death (cardiovascular, neurological, multi-organ failure) will be applied as hypothesis generating. Any changes from the pre-specified analysis plan will be reported.

#### Statistical software

Data will be analyzed using the latest versions of SAS® Enterprise Guide® (currently v7.1) and SAS Studio provided on Capital Region computers and servers (SAS-Institute Inc., Cary, NC).

### Interim analyses {21b}

As this is a pilot trial with a limited number of planned inclusions, no interim analysis will be performed; however, the sponsor-investigator will closely monitor the general safety of the trial participants.

### Methods for additional analyses (e.g., subgroup analyses) {20b}

With respect to analyses of whether IL-6RA reduces infarction size after ST-elevation myocardial infarction, this will only be investigated in a subgroup of patients with an acute coronary angiogram indicating this condition.

Also, the interaction of time to ROSC and time to infusion of the study drug on the changes in markers of inflammation compared to placebo will be investigated; for this, patients will be divided into two groups, below/above the median time to ROSC and time to infusion.

Further, the effects on the coagulation system by the study drug compared to placebo will be investigated by daily fibrinogen measurements and thrombelastography at admission and at 48 h; specifically, we will investigate if these differences between active and placebo are more pronounced in patients with hyperfibrinolysis.

### Methods in analysis to handle protocol non-adherence and any statistical methods to handle missing data {20c}

All analyses will be made on the modified intention to treat population, i.e., randomized patients for whom the study drug has been prepared and the patients next of kin has not refused participation when informed of the study and asked for consent.

As listed above, missingness of greater than 5% for the primary outcome (hsCRP) will prompt the use of multiple imputation.

### Plans to give access to the full protocol, participant-level data, and statistical code {31c}

The full protocol will be available upon reasonable request. Participant-level data will be made available upon reasonable request after full publication and approvals from relevant authorities.

## Oversight and monitoring

### Composition of the coordinating center and trial steering committee {5d}

The IMICA trial is a single-center study, initiated and overseen by the sponsor-investigator. See below for further details.

### Composition of the data monitoring committee, its role, and reporting structure {21a}

Yearly safety rapports will be filed to the Local Ethical Committee and Danish Medicines Agency, and patient safety will be monitored closely by the sponsor-investigator and co-investigators; however, as this is a trial of limited size, no data monitoring committee will be formed.

### Adverse event reporting and harms {22}

Adverse events will be assessed daily for the first 7 days, and adverse events occurring after day 7 will be evaluated at 30- and 180-day follow-up. At each assessment of all adverse events, serious adverse events (SAE) and suspected unexpected serious adverse reactions (SUSAR) must be recorded by the investigator and evaluated. Each SAE and SUSAR requires the sponsor to fill in the AE form, including the following variables: description of event, onset and end of event, severity, relation to the intervention, action taken, and outcome. Any adverse events occurring during the study will be treated according to established standards, and the subject will be followed until the event has disappeared or stabilized. On a yearly basis, a safety rapport containing information on serious adverse events/reactions will be submitted to the Danish Health and Medicines Authority.

An SAE will be defined as any AE that results in death, is life-threatening, requires prolongation of hospitalization, or results in significant disability, including congenital anomaly or birth defect.

For each AE, the investigator assesses potential causality between investigational products, and whether the reaction is suspected will be assessed by the sponsor. The Summary of Product Characteristics for RoActemra will be used for this assessment. The sponsor will be responsible to report all life-threatening lethal SUSARS to the Danish Health and Medicines Authority as soon as possible and no later than 7 days after being aware of such an event. Non-life-threatening SUSARs will be reported as soon as possible and no later than 15 days after the event.

### Frequency and plans for auditing trial conduct {23}

The trial will be externally monitored by the national Good Clinical Practice (GCP) unit at the Copenhagen GCP center. A monitoring plan will be conducted prior to trial initiation. The frequency of onsite monitoring will depend on compliance with the protocol, number of enrolled patients, and quality of data handling. There will be mandatory monitoring before and after the trial and at least once during the trial. The GCP will monitor inclusion and exclusion criteria, consent obtained in all subjects according to legislation, and data included in the eCRF. The principal investigator will be responsible for all data in the eCRFs.

### Plans for communicating important protocol amendments to relevant parties (e.g., trial participants, ethical committees) {25}

Major protocol modifications and related changes to patient information will only be implemented after approval from regulating authorities as appropriate.

### Dissemination plans {31a}

The results of the trial will be submitted for publication in international peer-reviewed journals and submitted for presentation at international conferences. Patients and their next of kin are asked at the time of consent if they would like to receive the results of the trial when completed.

## Discussion

In this study, we aim to reduce the biological effects of circulating IL-6 by administering tocilizumab at an early timepoint after OHCA to possibly dampen the systemic inflammatory response seen after OHCA, and the trial has been powered to detect a 30% reduction in hsCRP 48 h after OHCA. If this primary endpoint is achieved, we will investigate possible downstream effects of a reduced systemic inflammatory response on hemodynamic and organ function.

Whether previous findings of an association between higher levels of IL-6 is associated with morbidity and mortality [8, 9, 28, 29] predominantly reflects the nature of the resuscitation attempt or constitutes a causal component in triggering development of PCAS is unknown. However, mitigating the inflammatory response at this early stage might modify the severity of PCAS in turn possibly leading to less vasoplegia/decreased need of vasopressor support, more normal CO and SVR findings, and organ function.

As previously described, the use of IL6-RA in NSTEMI patients, at a dosage considerably lesser than in the present trial, reduced hsCRP and troponin levels [21], as well as altered cytokine levels [30]. The degree of systemic inflammation in these NSTEMI patients was much lesser than observed after OHCA and likely the effects of IL-6RA will be of a different magnitude in the present trial; likewise, a substantial proportion of OHCA patients in this trial will present with STEMI, again leading to higher levels of myocardial injury and troponin release. Inspired by these findings, we will be conducting an extensive measurement of a wide range of both pro-inflammatory and anti-inflammatory cytokines to possibly reveal a change in cytokine patterns following pharmaceutical blockage of the IL-6 receptor, as well as investigate whether IL-6RA leads to lessened troponin release in OHCA patients, including those with myocardial infarction.

Infection after OHCA is not uncommon [31–34], including in cases of presumed cardiac cause. IL6-RA has previously been shown to increase the frequency of infections in a randomized trial in rheumatoid arthritis [35]. Patients in IMICA will be monitored for infections, including sampling of tracheal secretion early after admission, and will be treated with prophylactic antibiotics as part of routine therapy (see the “Relevant concomitant care permitted or prohibited during the trial {11d}” section).

CRP was chosen as the primary endpoint, in solitude, since CRP levels were considered to perceptively reflect the biologic effects of circulating IL-6 (as IL-6 regulates CRP production) [13], i.e., if CRP production was substantially lessened, we could assume that the dosage of tocilizumab would also be sufficient to allow for other IL-6-mediated effects to be present in the active treatment group—namely any of the secondary endpoints. As this study is to be considered a pilot study, we chose to not decide on a co-primary endpoint from among the present secondary endpoints, as many of these would seem as equally important clinically benefits to target in a future larger trial. If results from the present trial indicate that a substantial clinical benefit could be expected from IL-6 receptor blockage after OHCA, a future trial should be designed and powered to detect possible increased chances of survival with a good neurological outcome. Only such a trial, and not the present of limited size, should prompt changes in treatment guidelines.

## Trial status

Protocol Version: v1.91 of November 1, 2019 (Minor updates to v1.9 of October 25, 2018)

Recruiting; first patient included on March 4, 2019. Estimated recruitment completed before March 2020 and completion of 180-day follow-up before September 2020.

## Abbreviations

ABG: Arterial blood gas
AE: Adverse event
CO: Cardiac output
CVP: Central venous pressure
ECG: Electrocardiogram
eGFR: Estimated glomerular filtration rate
G-CSF: Granulocyte-colony stimulating factor
GM-CSF: Granulocyte-macrophage colony-stimulating factor
HR: Heart rate
IL: Interleukin
INF-g: Interferon-gamma
LVEF: Left ventricular ejection fraction
MAP: Mean arterial blood pressure
MCP-1: Monocyte chemoattractant protein-1
MIP-1beta: Macrophage inflammatory protein-1beta
MOCA: Montreal Cognitive Assessment
MR: Magnetic resonance imaging
NSTEMI: Non-ST elevation myocardial infarction
PCWP: Pulmonary capillary wedge pressure
ROSC: Return of spontaneous circulation
SAE: Serious adverse event
SOFA: Sequential organ failure assessment
STEMI: ST elevation myocardial infarction
SUSAR: Suspected unexpected adverse reaction
SVR: Systemic vascular resistance
TAPSE: Tricuspid annular plane systolic excursion
TEG: Thrombelastography
TNF-alfa: Tumor necrosis factor-alfa
TNT: Troponin T
VBG: Venous blood gas
OHCA: Out-of-hospital cardiac arrest
PACS: Post-cardiac arrest syndrome
